# Identification and Validation of FGF-Related Prognostic Signatures in Prostate Cancer

**DOI:** 10.1155/2023/7342882

**Published:** 2023-02-21

**Authors:** Yongkang Ye, Rujun Mo, Ruinian Zheng, Jun Zou, Shaoqian Liu, Qiwu Mi, Weide Zhong

**Affiliations:** ^1^Department of Urology, Dongguan People's Hospital, Affiliated to Southern Medical University, Dongguan, China; ^2^Dongguan Institute of Clinical Cancer Research, Department of Medical Oncology, Affiliated to Dongguan Hospital, Southern Medical University (Dongguan People's Hospital), Dongguan, China; ^3^Department of Emergency Surgery, The Third Affiliated Hospital of Guangzhou Medical University, Guangzhou, China; ^4^Department of Urology, Guangzhou First People's Hospital, South China University of Technology, Guangzhou, China

## Abstract

**Background:**

FGF signaling is critical to controlling various cancers. Nevertheless, the functions of FGF-related genes in PCa are still unknown.

**Objective:**

The objective of this study is to build a FGF-related signature that was capable of accurately predicting PCa survival and prognosis for BCR.

**Methods:**

The univariate and multivariate Cox regression, infiltrating immune cells, LASSO, and GSEA analyses were carried out to build a prognostic model.

**Results:**

A FGF-related signature that consists of PIK3CA and SOS1 was developed for the purpose of predicting PCa prognosis, and all patients were categorized into low- and high-risk groups. In comparison to the low-risk group, high-risk score patients had poorer BCR survival. This signature's predictive power has been investigated utilizing the AUC of the ROC curves. The risk score has been shown to be an independent prognostic factor by multivariate analysis. The four enriched pathways of the high-risk group were obtained by gene set enrichment analysis (GSEA) and found to be associated with the tumorigenesis and development of PCa, including focal adhesion, TGF-*β* signaling pathway, adherens junction, and ECM receptor interaction. The high-risk groups had considerably higher levels of immune status and tumor immune cell infiltration, suggesting a more favorable response to immune checkpoint inhibitors. IHC found that the expression of the two FGF-related genes in the predictive signature was extremely different in PCa tissues.

**Conclusion:**

To summarize, our FGF-related risk signature may effectively predict and diagnose PCa, indicating that in PCa patients, they are potential therapeutic targets and promising prognostic biomarkers.

## 1. Introduction

Prostate cancer or PCa is considered the most prevalent malignancy in worldwide male adults [1]. Accounting for rapidly rising morbidity and mortality, PCa remains worrisome for the majority of countries [2]. In 2020, prostate cancer accounted 1,414,259 new cases and 375,304 associated deaths have occurred globally. By 2030, the number of new PCa cases worldwide is predicted to increase to 1,700,000 and lead to about 500,000 deaths. At present, the prostate cancer therapies are chemotherapy, surgical resection, radiotherapy, and hormone therapy. The main treatments of the clinic in advanced PCa are androgen deprivation therapy (ADT) and combination neoadjuvant hormone therapy (NHT) [3, 4]. Nearly 20 percent of patients may experience clinical recurrence after radical prostatectomy (RP), defined biochemical recurrence (BCR), which was considerably linked to tumor specific death, overall risk of death, and distant metastasis. After RP, monitoring BCR for patients with prostate cancer (pathological staging, serum, grouping, and interval from radical treatment to biochemical recurrence) was very important for subsequent treatments. And a retrospective study indicated that the BCR survival rates at 1-, 5-, and 10-year were 0.981, 0.831, and 0.684, respectively. Hence, identifying novel prognostic biomarkers based on BCR survival is critical, making targeted treatments highly feasible for prostate cancer.

Fibroblast growth factors (FGFs) are a big family of secreted proteins, including 18 FGF ligands and 4 FGF receptors (FGFRs) [5]. FGFs binding to their cognate FGFRs have been demonstrated to activate a number of signaling cascades, consisting of phospholipase C gamma (PLC-*γ*), phosphatidylinositol-3 kinase (PI3K), and Ras to extracellular signal-regulated protein kinase (ERK) pathways [6]. FGF signaling is crucial to regulating a number of cellular processes, which consist of stemness, metabolism, proliferation, angiogenesis, epithelial-mesenchymal transition, and immunity through its receptors [7–11]. A significant amount of convincing evidence for a critical role of FGFR signaling in the progression of cancers, such as breast, lung, prostate, pancreas, bladder, cervical, and ovarian, has been collected by oncological researchers, and in these tumor types, the FGFRs can act as molecular targets for specific anticancer therapy. For instance, Mahmood et al. reported that treatment with the FGFR inhibitor decreased the expression of the HPV 16/18 E7 oncoprotein that most likely is responsible for the malignant transformation and cervical tumor development [12]. In terms of prostate cancer, dysregulation of FGFR signaling has been widely reported. Yim et al. reported that casein kinase 1 (CK1) inhibition decreased the FGF-ERK signaling via inhibiting SPRY2 for neurite outgrowth in PC12 cells [13]. Primary human dermal fibroblasts with increased FGF signaling have been indicated to promote macrophage infiltration [14]. The role of FGFs binding to their receptors induced signaling pathway in PCa, which represents a related regulator of tumor-stroma interaction, cancer cells growth, metastasis, and recurrence, particularly after androgen deprivation therapy (ADT). Bluemn et al.'s study revealed that both androgen receptor (AR) and neuroendocrine null PCa are notable for increased FGF and MAPK signaling pathways, and then FGF/MAPK blockade inhibited PCa growth in metastatic PCa with an AR-null phenotype [15]. LIM domain only 2 (LMO2) was put forward as a new marker in prostate cancer, which increases the viability of prostate cancer cells via releasing FGF-9 and IL-11 from prostate fibroblasts [16]. Chiodelli et al. reported that FGFR inhibitor pemigatinib alone or in combination with enzalutamide causes intracellular stress as well as cell death in naive and castration-resistant prostate cancer [17]. Hence, the related genes of the FGF signaling pathway (FGF-related) are emerging as promising targets for the treatment of PCa.

**Figure 1:**
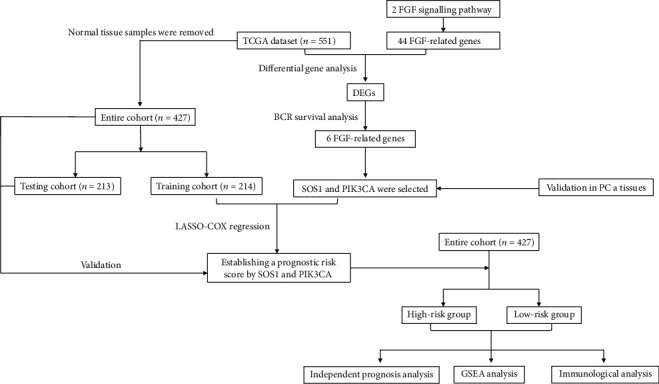
Flow chart of the whole study.

In our research, we aim to devise a novel prognostic signature on the basis of the FGF-related genes for the purpose of investigating the correlation among clinicopathological features and signature and BCR from the TCGA database. Then, we analyzed biological functions and enriched pathways and assessed their differential expression in various risk samples of prostate cancer. After that, we chose SOS1and PIK3CA for further validation in vitro. Moreover, in the TCGA dataset, a prognostic model based on FGFs was also created. The detailed workflow in our study is shown in Figure 1.

## 2. Materials and Methods

### 2.1. Data Download

The Cancer Genome Atlas (TCGA) website (https://portal.gdc.cancer.gov/repository) was the source of the RNA-seq (FPKM value) data of prostate cancer and the corresponding clinical data for 499 prostate cancer tissue samples as well as 52 normal prostate tissues.

### 2.2. FGF-Related Protein-Protein Interaction (PPI) Network Construction

The association between candidate proteins was analyzed using a PPI network website (https://string-db.org/cgi/network). And Cytoscape (version 3.7.1) was used to discern the PPI networks between the candidate proteins [18].

### 2.3. Consensus Clustering of FGF-Related Genes

Utilizing the “ConsensusClusterPlus” package of the R, numbers of clusters were determined according to the following criteria: relatively high consistency within the cluster, relatively low coefficient of variation, and no appreciable increase in the area under the CDF curve, and individuals with prostate cancer were separated into various subgroups on the basis of the FGF-related genes [19].

### 2.4. Development and Validation of the FGF-Related Signature

In this study, 44 genes in total were examined (Supplementary Table S1). Employing *P* values < 0.05 as the cut-off, univariate Cox was carried out to identify genes associated with biochemical recurrence-free survival (BCR-free). The risk of overfitting was reduced by using the LASSO approach. Finally, the risk model was developed using multiple stepwise Cox regression. (exprgene1 × coefficientgene1) + (exprgene2 × coefficientgene2) are described as the hub gene's risk score. In addition, both univariate and multivariate Cox regression analyses were performed using clinical features and the risk scores to assess the independent prognostic value of the signature.

### 2.5. Functional Enrichment Analysis

The “Consensus ClusterPlus” package was used to achieve FGF-based consensus clustering. KEGG and GO analyses on the basis of the differentially expressed genes (DEGs) (|log2FC| ≥ 1, FDR < 0.05) among various risk groups were performed using the “clusterProfiler” R package. Twenty-eight cell filtrating score was computed with ssGSEA in the “GSVA” R package. Supplementary Table S2 displays the file for the annotated gene set. In TCGA individuals, the cBioPortal was the source of the genetic variations of specific genes.

### 2.6. Evaluation of Infiltrating Immune Cells

We determined the immune cell proportions of 22 kinds of infiltrating immune cells in PCa transcriptional profiles via the CIBERSORT web portal (https://cibersort.stanford.edu/). The disparity in the immune cell infiltration level was evaluated in the low- as well as high-risk groups with the criteria of *P* < 0.05.

### 2.7. Immunohistochemistry

Expression pattern and subcellular localization of SOS1 (HUABIO, HA720077, 1 : 400) and PIK3CA (HUABIO, ET1606-36, 1 : 50) protein in clinical PCa tissues were detected by immunohistochemistry (IHC). Two tumor specimens (Bioaitech, M196Pr01) were scored in a semiquantitative manner. The scores of ICH staining in each case were performed in accordance with the percentages and the intensity.

### 2.8. Statistical Analysis

The R (v.3.6.1) software was utilized for all statistical investigations. The log-rank test was employed to examine the survival difference of BCR. Utilizing chi-square tests, *t*-tests, or nonparametric tests, differences across variables were examined. *P* < 0.05 was deemed statistically significant.

## 3. Results

### 3.1. Expression and Correlation of FGFs in the TCGA Cohort

**Figure 2:**
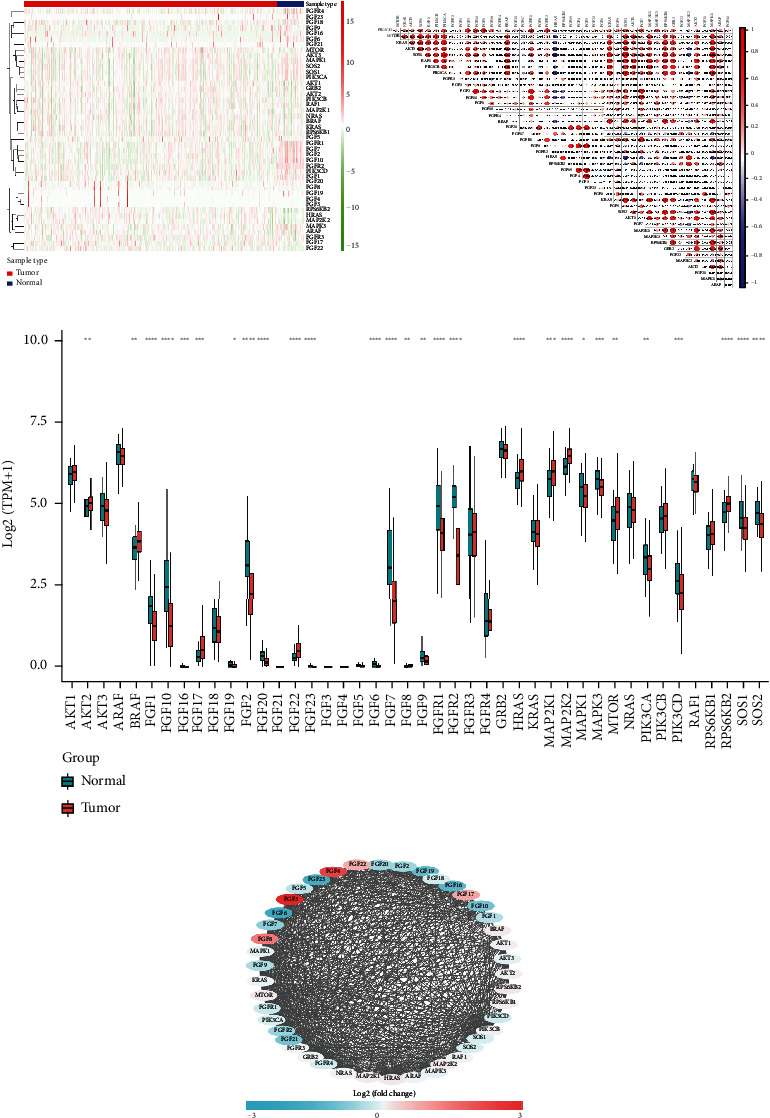
The landscape of FGF-related genes in prostate cancer. (a) Heat map of FGF-related genes in normal samples and PCa samples from TCGA database (the right ordinate represents differential gene names; the left dendrogram represents differential gene expression clustering; each small square represents the expression of a gene in one sample, and the upper right histogram is color scale. (b) Pearson correlation analysis of FGF-related genes (the number in each circle in the figure indicates the correlation coefficient between the two genes, and the ∘ marker indicates that the two genes are not significantly correlated). (c) Box plot of the expression of FGF-related genes in normal control samples and PCa samples (abscissa indicates gene name and ordinate indicates gene expression value; blue is for normal samples and red for HNSCC samples). (d) The protein-protein interaction network of FGF-related genes (each node in the figure represents a protein and the edge between the nodes represents the interaction between the two proteins). Red circles represent the protein products of upregulated genes; green circles represent the protein products of downregulated genes. The smaller the *P* value after correction is, the more significant the difference is.

In the TCGA dataset, the RNA expression of FGFs was examined. Most differentially expressed FGFs (10/44, 44%) were identified in tumor samples in comparison to normal tissues (Figures 2(a) and 2(c)). Among them, 10 were upregulated and 5 were downregulated in the prostate cancer tumors. The heat map exhibits the RNA expression of 44 FGFs. In addition, there was more interaction network among these 44 genes (Figure 2(d)). Furthermore, the association among these genes has been assessed (Figure 2(b)); for instance, PIK3CA was considerably positively related to SOS1 (Cor = 0.9), and FGF4 was considerably positively related to FGF3 (Cor = 0.94), while SOS1 and HRAS were negatively correlated (Cor = −0.61).

### 3.2. Identification of BCR-Free-Related FGFs

**Figure 3:**
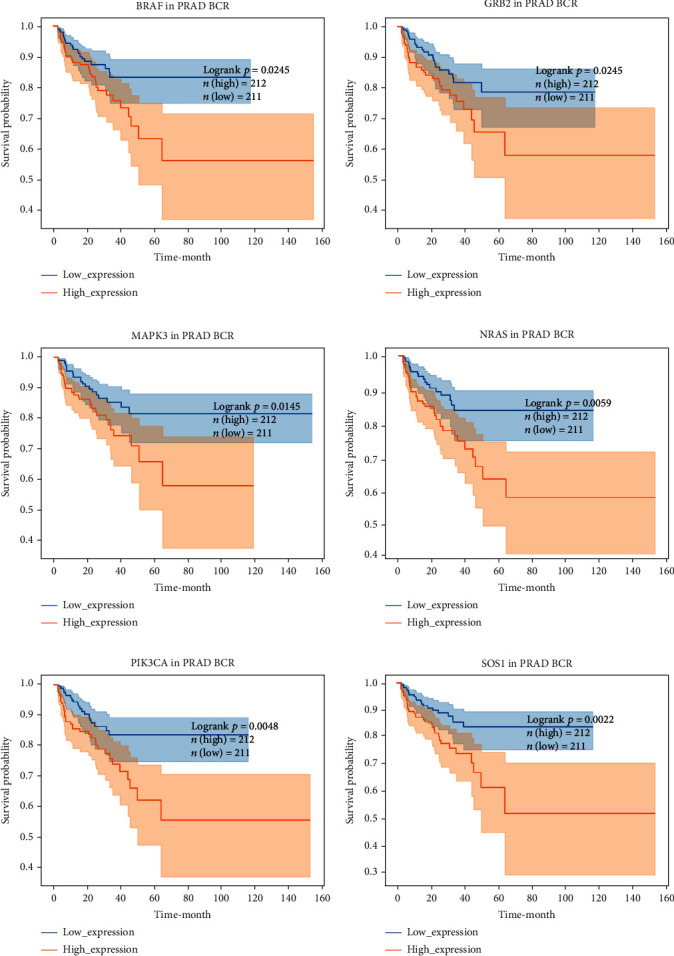
Kaplan-Meier survival curve analysis of the association between six prognostic marker genes. (a)–(f) Kaplan-Meier survival curves for the association of six prognostic marker genes (PIK3CA, GRB2, SOS1, NRAS, BRAF, and MAPK3) with the prognosis of PCa patients (the abscissa indicates survival time, the ordinate indicates survival rate, the orange line indicates patient survival status of high expression genes, and the blue line indicates patient survival status of the low expression group).

For further determining FGFs with the highest prognostic value, we performed biochemical recurrence-free (BCR-free) survival outcome by Kaplan-Meier (K-M) analysis. 6 of the 44 FGFs with high expression (PIK3CA, GRB2, SOS1, NRAS, BRAF, and MAPK3) in PCa patients were found to be associated with PCa BCR in comparison to its low expression (*P* = 0.0048, *P* = 0.0245, *P* = 0.0022, *P* = 0.0059, *P* = 0.0245, and *P* = 0.0145, respectively, Figure 3), and then, we choose two hub FRGs (SOS1 and PIK3CA) to validate by TMA analysis and construct a risk signature of PCa patients.

### 3.3. Expression of SOS1 in PCa Tissue

**Figure 4:**
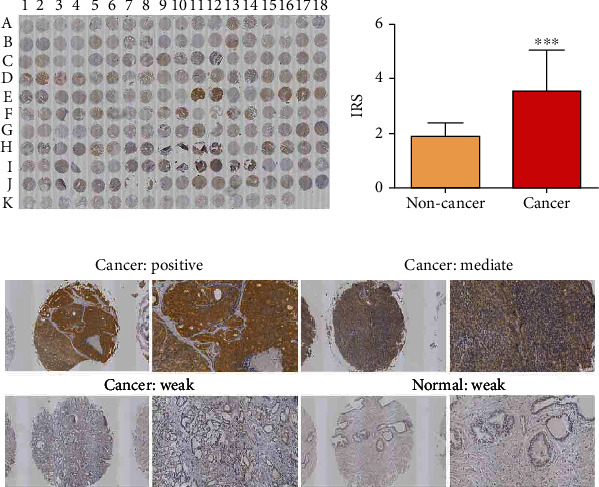
Immunohistochemical staining for PIK3CA in PCa and normal prostate tissues in TMA samples. (a) Full view of the immunohistochemistry staining for PIK3CA. (b) Expression level of PIK3CA in PCa tissues was significantly higher than in normal prostate tissues. (c) Immunostaining of PIK3CA high, medium, and low expression.

For assessing the impact of SOS1 in prostates of PCa patients, we measured the proteins of SOS1 in PCa as well as noncancer tissues via immunohistochemical analysis. The SOS1 protein expression was determined as low (immunoreactivity score (IRS): 0-4) and high (IRS: 4-8). As depicted in Figures 4(a) and 4(c), SOS1 protein expressions were detected at high levels in the cytoplasm and membranous in PCa tissues, whereas they occurred at low levels in noncancer tissues. The expression of SOS1 proteins in PCa tissues was markedly upregulated, in contrast to that of non-PCa tissue (*P* < 0.001, Figure 4(b)).

### 3.4. Expression of PIK3CA in PCa Tissue

**Figure 5:**
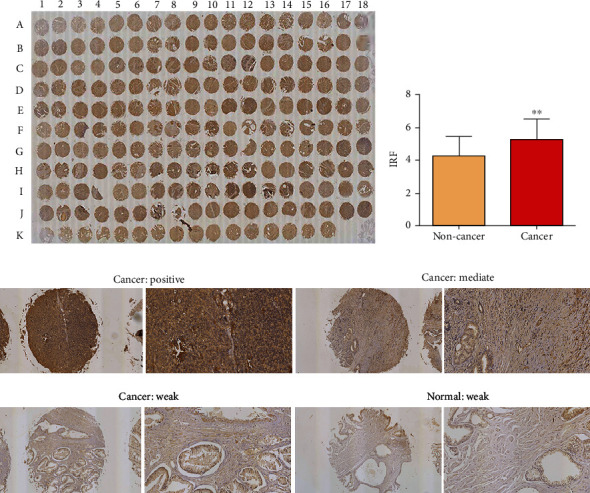
Immunohistochemical staining for SOS1 in PCa and normal prostate tissues in TMA samples. (a) Full view of the immunohistochemistry staining for SOS1. (b) Expression level of SOS1 in PCa tissues was significantly higher than in normal prostate tissues. (c) Immunostaining of SOS1 high, medium, and low expression.

For investigating the impact of PIK3CA in prostates of PCa patients, the proteins of PIK3CA were measured in PCa and noncancer tissues via immunohistochemical analysis. The PIK3CA protein expression levels were determined as low (immunoreactivity score (IRS): 0-4) and high (IRS: 4-8). Figures 4(c) and 5(a) highlight that PIK3CA protein expression was detected at high levels in the cytoplasm and membranous in PCa tissues, even though they occurred at intermediate levels in non-PCa tissues. When compared to non-PCa tissue (*P* < 0.01, Figure 5(b)), the expression of PIK3CA proteins in PCa tissues was markedly upregulated.

### 3.5. Construction of a Two-FGF Prognostic Model

**Figure 6:**
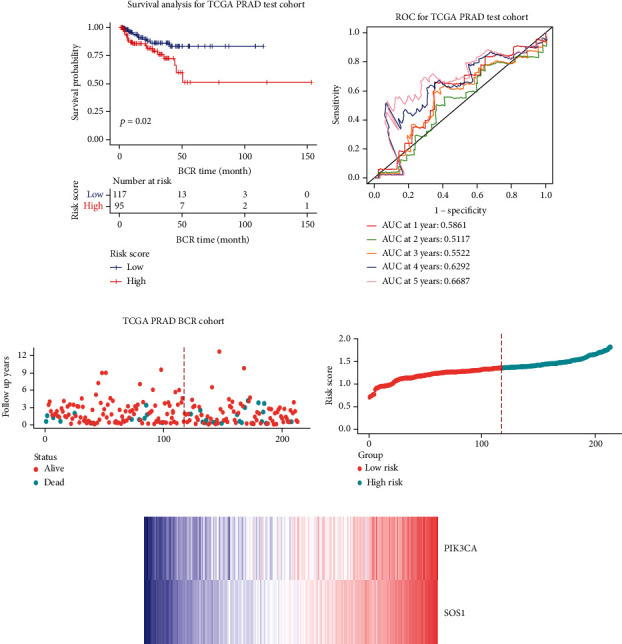
Development of the prognostic signature based on PIK3CA and SOS1 in the training set. (a) Kaplan-Meier (KM) curves of the BCR in the training cohort. (b) ROC curves and area under the curve (AUC) for 1-, 2-, 3-, 4-, and 5-year survival in the training cohort of the risk model. (c)‑(e) Distribution of risk score, OS, and survival status (red dots indicate death; blue dots indicate alive) and the two genes' expression heat map in the training cohort.

The whole cohort of 427 tumor samples was divided into two groups at random: a testing cohort (*n* = 213) and a training cohort (*n* = 214). Through following procedures, each PCa patient's risk scores were measured: Risk score = (0.096^∗^ExpSOS1) + (0.247^∗^ExpPIK3CA). Then, based on the risk score's median value (1.3564) in a training cohort, we categorized the prostate cancer patients into the low-risk (*n* = 107) and high-risk (*n* = 107) groups. In accordance with the K-M plot, the high-risk group had a poor survival prospect in comparison to the low-risk group (*P* = 0.009, Figure 6(a)). Additionally, the area under the receiver operating characteristic curve (AUC) for the two-FGF prognostic model at 1-, 2-, 3-, 4-, and 5-year BCR-free were 0.6194, 0.5889, 0.5624, 0.5624, and 0.5624 (Figure 6(b)), respectively, suggested good classification findings. The heat map, survival status, and risk score distribution reveal that the risk model could predict the prognosis of prostate cancer patients accurately (Figures 6(c)–6(e)).

### 3.6. Validation of the Prognostic Model

**Figure 7:**
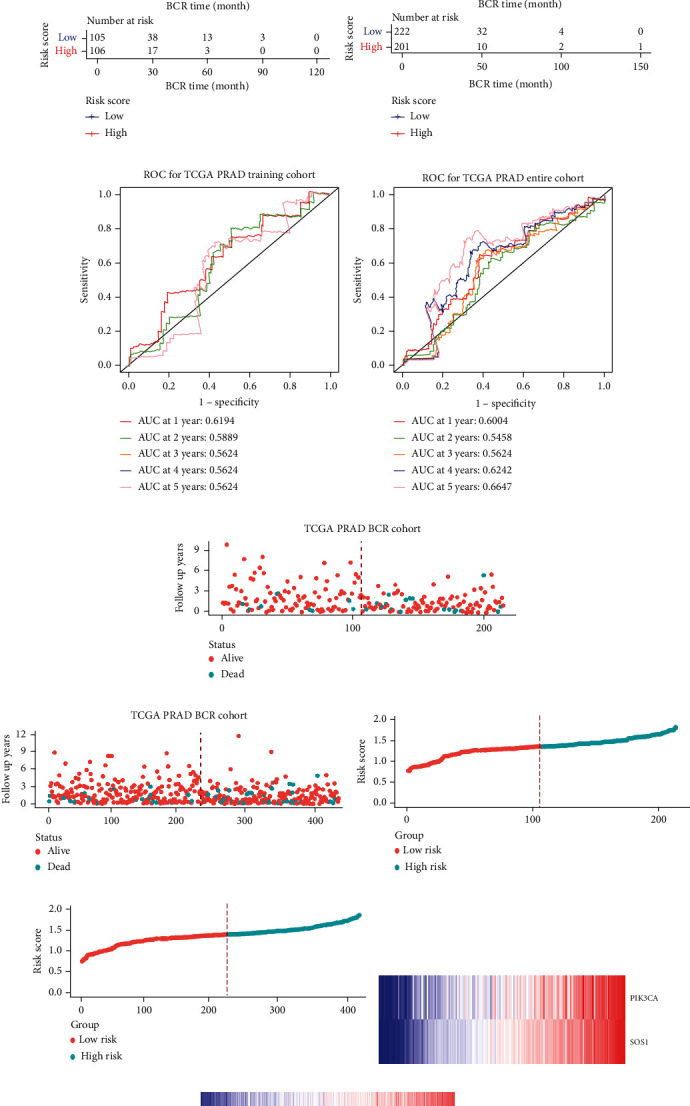
Validation of the prognostic signature in the testing set and the total set. (a, b) The survival curve showed the different BCR status between high- and low-risk patients in the testing set and the total set. (c, d) Receiver operating characteristic curves of prognostic signature in the testing set and the total set. (e, f) The scatter plot showed the BCR status of PCa patients in the testing set and the total set. (g, h) The risk curve of each sample reordered by risk score in the testing set and the total set. (i, j) The heat map showed the expression of prognostic genes in the testing set and the total set.

We examined the risk model in both the entire TCGA cohort and testing cohort to confirm its precision. Each patient participating in the entire cohort and testing had their risk score determined, which is then separated into two groups on the basis of the median. 95 and 118 patients from the testing cohort were separated into the high- and low-risk groups, respectively. Similar to this, 225 and 202 individuals were categorized into the high- and low-risk groups, respectively. Considerable differences exist in survival curves amongst two risk groups in the entire cohort (*P* = 0.00042, Figure 7(b)) and the testing cohort (*P* = 0.02, Figure 7(a)). The AUC values at 1-, 2-, 3-, 4-, and 5-year in the testing cohort were 0.5861, 0.5117, 0.5522, 0.6292, and 0.6687, respectively (Figure 7(c)) in the entire cohort, the AUC values at 1-, 2-, 3-, 4-, and 5-year were 0.6004, 0.5458, 0.5624, 0.6242, and 0.6647, respectively (Figure 7(d)). In the two cohorts, heat map, the risk score distribution plot, and survival status plot of risk gene expression were displayed (Figures 7(e)–7(j)). The level of each risk gene was more in the high-risk group than the low-risk group, demonstrating the ability of the risk model to predict the prognosis of individuals with prostate cancer.

### 3.7. Independent Prognostic Value of the Risk Model

The univariate Cox regression analysis revealed that in the entire cohort, the Gleason score (HR = 3.84, 95% CI: 2.13-6.91, *P* value < 0.001), risk score (HR = 2.56, 95% CI: 1.49-4.38, *P* value = 0.0007), lymph node (HR = 1.91, 95% CI: 1.07-3.42, *P* value = 0.03), tumor stage (HR = 5.03, 95% CI: 2.16-11.74, *P* value = 0.0002), and PSA (HR = 9.34, 95% CI: 3.95-22.06, *P* value < 0.001) were related to BCR-free, and the risk score, Gleason score, tumor stage, and PSA remained independent predictors via multivariate Cox regression analysis (Table 1). These outcomes pointed out that PSA, the risk score, and tumor stage could be independent predictors of BCR-free survival of PCa patients.

### 3.8. Functional Enrichment Analysis of Genes Correlated with Signature Genes

We performed ssGSEA on the high-risk group and the low-risk group to further explore the significantly enriched pathways. The results showed that 163 KEGG enriched pathways were active in the high-risk group (Supplementary Table S2_1) and 14 were active in the low-risk group (Supplementary Table S2_2). Furthermore, based on *P* value of <0.05 and FDR of <0.25 were considered statistically significant, 15 statistically significant KEGG pathways were screened, 13 were active in the high-risk group, and 2 were active in the low-risk group among them (Table 2).

### 3.9. Association among Immune Cell Infiltration and the Risk Model

**Figure 8:**
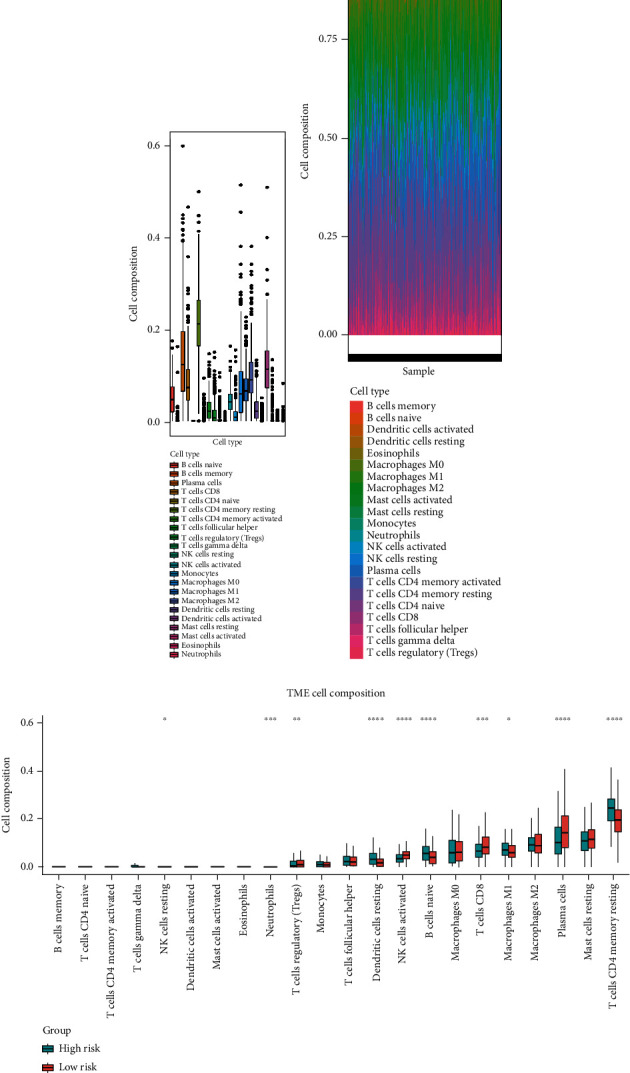
Infiltrating tumoral immune cell composition analysis. (a, b) The proportion of 22 immune cell types in PCa from the TCGA datasets. (c) The boxplot shows the different infiltration levels of 22 immune cell types between the high and low risk expressions. ^∗^*P* < 0.05, ^∗∗^*P* < 0.01, ^∗∗∗^*P* < 0.001, and ^∗∗∗∗^*P* < 0.0001.

To investigate the correlation between immune cell infiltration and the prognostic signature, we used the CIBERSORT algorithm to calculate the content of 22 immune cell populations in each PCa sample by setting *P* < 0.05 as the threshold for screening. The results showed that T cells CD4 naïve were the most abundant infiltrating cells, followed by plasma cell and mast cell resting (Figures 8(a) and 8(b)). We also observed differences in immune cell infiltration between the two risk groups. As the results showed in Figure 8(c), the content of NK cell resting, dendritic cell resting, B cell naive, T cell CD4 memory resting cells, and macrophages M1 and risk score was positively associated in prostate cancer tissues (*P* < 0.05). The outcome showed that in prostate cancer patients, the tumor immune microenvironment state may be reflected by the gene model.

## 4. Discussion

With the advancement of gene chips and high-throughput second-generation sequencing technologies, the amount of publicly available high-throughput data is stored in global databases. Based on the GEO and TCGA databases, some studies have reported to construct predictive models and identify new signatures so as to diagnose or predict the survival of PCa in the recent years. Li et al. identified two lncRNA, MKNK1-AS1 and INE1a, as candidates for the prognostic signature based on the TCGA-PRAD and GEO datasets, which are associated with autophagy regulation in PCa [19]. Six immune-related signatures were discovered and verified by Luan et al. as independent predictive variables for BCR in prostate cancer [20]. Recent evidence suggests that aberrant activation of FGF/FGFR signaling is closely connected to tumorigenesis, because of constitutive activation of multiple downstream pathways, for instance, mitogen-activated protein kinase and phosphoinositide 3-kinase. Thus, to make targeted therapies more feasible in the future for BCR, we used machine learning to analyze data on a large number of PCa patients and established a prognostic model based on a panel of 2 FGF-related genes and clinical characteristics. The model was established and validated using the TCGA database, and it showed good discrimination and calibration in predicting survival. Finally, the connection between the model and immunity as its mechanistic basis has been partially confirmed.

Several studies have previously revealed that dysregulated FGF-related signaling causes a wide range of human disorders. Through differential gene analysis of PCa patients, the mRNA expression patterns of 44 FGF-related genes in TCGA PCa, as well as normal samples, were examined in this study, with 10 being differentially expressed. Six of these genes were associated with the survival of BCR, and two genes were selected after LASSO Cox regression analyses. Meanwhile, we also constructed a prognostic model and illustrated the impact of the expression levels of these two genes on the BCR of PCa patients, based on a combination of the risk score distribution, survival status scatter plot, and gene expression heat map, which provided a robust indicator for the prognostic evaluation of PCa. Luckily, these genes have been confirmed to be closely related to cancer. PIK3CA act as an alpha catalytic subunit of phosphatidylinositol 3-kinase (PI3K), the activating mutations of the PIK3CA gene, which include prostate colorectal, head and neck, and breast cancers [21–23]. Herberts et al. have demonstrated that 6% of advanced prostate cancer patients have activating mutations in the genes PIK3CA. This may help in selecting patients for clinical trials of PI3K pathway inhibitors [24]. Intermittent treatment of BAY1082439A, a selective PI3K inhibitor against the PI3K*α*/*β*/*δ* isoforms, can overcome resistance to immune checkpoint therapy in the PTEN-null model [25]. SOS1 is an activator of Ras/mitogen-activated protein kinase (MAPK) and was overexpressed in African-American men's prostate cancer epithelial cells, which enhanced their chance of developing PCa [26]. Lin et al. had identified casticin inhibition of PCa cell migration and invasion and downregulation of the expression of SOS1, and the findings may represent a promising therapeutic agent for metastatic PCa about SOS1 [27]. In our study, it has also been observed that the expression of PIK3CA and SOS1 was upregulated via IHC and associated with a shorter BCR in PCa patients. These results may illustrate that interfering with the expression of PIK3CA and SOS1 may affect the therapy of prostate cancer to a certain extent.

Additionally, the clinical characteristics of PCa patients are closely related to prognosis. To better optimize the model and improve the accuracy of patient BCR survival prediction, clinical information in TCGA (including Gleason score, TNM stage, PSA, and lymph node stage) was selected through univariate and multivariate Cox analysis. TNM stage is an important criterion for the current staging of cancer patients and is widely considered to be the standard approach for predicting prognosis in most solid tumors [28, 29]. Furthermore, PSA, lymph node stage, and Gleason score are the most frequent markers for prostate cancer screening, diagnosis, and prognosis evaluation in predicting the final pathologic stage all over the world [30–32]. Our study indicated that risk score could be used as an independent prognostic factor to predict the prognosis of patients. In terms of clinical relevance, the prognostic signature was significantly correlated with Gleason score, lymph node stage, tumor stage, and PSA. Therefore, independent prognostic value of the risk model that had a good prognosis prediction of BCR was built in the study,

Although many studies have demonstrated the association of aberrant FGFs with the development and progression of PCa [33, 34], the mechanisms of FGFs in PCa are not yet clear. In order to elucidate the mechanisms underlying the signature, the enriched pathways in the high- and low-risk group obtained by GSEA analysis were significantly associated with the TGF-*β* signaling pathway, which had been extensively confirmed to play a key role not only in PCa but also in neurological disorders, cardiovascular disease, gastric cancer, breast cancer, and lung cancer [6, 35–37]. Besides, we found that the signature expression showed a robust correlation with focal adhesion, adherens junction, and ECM receptor interaction, suggesting that the signature might promote the FGF-related signaling process through these pathways.

After that, the immune microenvironment is associated with the occurrence and development of PCa. In this study, our prognostic signature can further stratify PCa patients into subgroups with different immune infiltrations which indicated that numerous immune cells, such as the M1 phenotype of macrophages, NK cell resting, dendritic cell resting, T cell CD4 memory resting, and B cell naïve, were significantly higher in the high-risk group than that in the low-risk group. Patients in the high-risk group had a worst prognosis in our study. Meanwhile, we also found that the distribution of NK cell resting was the most significant difference between the high- and low-risk group, and the suggested risk scores were positively correlated with NK cell inhibition in TME of PCa. Previous studies have shown that primitive NK cells are derived from bone marrow lymphoid stem cells and are in a resting state in the peripheral circulation. NK cell resting became activated after stimulation with cytokines such as IL-2 to activate the MAPK pathway [38]. The activated NK cells can kill tumor cells nonspecific without presensitization [39]. Therefore, our data also revealed the importance of NK cell resting in the research of prostate cancer, which is a new discovery by the risk model and also consistent with the results of other tumor before. However, a large number of experiments are still needed whether the risk model is suitable for immunotherapy for further study.

There are still many limitations in our investigation. First, a TCGA PCa cohort was used to create and validate the risk model. Hence, more external validation cohorts and clinical data from PCa patients are needed to validate the signature's predictive power in PCa. Second, although we found the signatures including SOS1 and PIK3CA play different roles in the FGF-related signaling process through other important cancer-related signaling pathways and immunotherapy-related NK cells for PCa patients, some basic experiments should be explored further, such as the biological functions and the specific mechanism of SOS1 and PIK3CA overexpression both in vivo and in vitro.

## 5. Conclusion

In conclusion, two prognostic signatures (SOS1 and PIK3CA) that were closely associated with the prognosis of PCa patients were screened based on the TCGA database. The prognostic risk model of two signatures was constructed, and the prognostic risk model could effectively assess the prognosis differences of PCa patients and improve prognosis of prostate cancer patients at a high-risk group for immunotherapy. Therefore, these two prognostic marker genes will be used as molecular markers for prognosis prediction of PCa patients and guiding effective immunotherapy methods.

## Figures and Tables

**Table 1:** Independent prognostic value of the risk model.

Variables	Univariate analysis			Multivariable analysis		
	HR	95% CI of HR	*P*	HR	95% CI of HR	*P*
Risk score						
Low risk	2.5558	1.4898-4.3847	0.0007	2.329	1.3117-4.135	0.0039
High risk						
Gleason score						
<8	3.8395	2.1328-6.912	0	2.156	1.0821-4.296	0.0289
≥8						
Lymph node stage						
N0	1.9119	1.0676-3.4239	0.0293	1.085	0.5873-2.003	0.7951
N1						
Tumor stage						
T1-T2	5.0389	2.1619-11.744	0.0002	3.065	1.1375-8.259	0.0268
T3-T4						
PSA						
<10	9.3373	3.9532-22.055	0	4.851	1.8673-12.603	0.0012
≥10						

**Table 2:** Functional enrichment analysis of genes correlated with signature genes in the high-risk and low-risk group via GSEA.

Name	NES	*P* value	FDR
High-risk group			
KEGG_SMALL_CELL_LUNG_CANCER	1.4441	0.001	0.2425
KEGG_DORSO_VENTRAL_AXIS_FORMATION	1.4392	0.0171	0.1969
KEGG_RENAL_CELL_CARCINOMA	1.4287	0	0.1827
KEGG_ECM_RECEPTOR_INTERACTION	1.4142	0	0.1889
KEGG_NON_SMALL_CELL_LUNG_CANCER	1.4075	0.001	0.1767
KEGG_INOSITOL_PHOSPHATE_METABOLISM	1.3939	0.004	0.188
KEGG_TGF_BETA_SIGNALING_PATHWAY	1.3892	0.001	0.1789
KEGG_FOCAL_ADHESION	1.3787	0	0.1865
KEGG_DILATED_CARDIOMYOPATHY	1.3691	0.002	0.1921
KEGG_MELANOMA	1.3509	0.004	0.2236
KEGG_REGULATION_OF_ACTIN_CYTOSKELETON	1.3489	0	0.212
KEGG_ADHERENS_JUNCTION	1.3475	0.004	0.2005
KEGG_PATHWAYS_IN_CANCER	1.324	0	0.2376
Low-risk group			
KEGG_PROTEIN_EXPORT	-1.4866	0	0.0171
KEGG_PORPHYRIN_AND_CHLOROPHYLL_METABOL	-1.0799	0	0.1878

## Data Availability

The original contributions presented in the study are included in the article. Further inquiries can be directed to the corresponding authors.

## Supplementary Materials

Supplementary Materials Table S1: the mRNA expression patterns of 44 FGF-related genes in TCGA PCa, as well as normal samples. Table S2_1: the enriched pathways in the high-risk group obtained by GSEA analysis. Table S2_2: the enriched pathways in the low-risk group obtained by GSEA analysis.
